# EnzyMine: a comprehensive database for enzyme function annotation with enzymatic reaction chemical feature

**DOI:** 10.1093/database/baaa065

**Published:** 2020-10-01

**Authors:** Dandan Sun, Xingxiang Cheng, Yu Tian, Shaozhen Ding, Dachuan Zhang, Pengli Cai, Qian-nan Hu

**Affiliations:** CAS Key Laboratory of Computational Biology, CAS-MPG Partner Institute for Computational Biology, Shanghai Institute of Nutrition and Health, University of Chinese Academy of Sciences, Chinese Academy of Sciences, Shanghai 200333, P. R. China; CAS Key Laboratory of Computational Biology, CAS-MPG Partner Institute for Computational Biology, Shanghai Institute of Nutrition and Health, University of Chinese Academy of Sciences, Chinese Academy of Sciences, Shanghai 200333, P. R. China; School of Biology and Pharmaceutical Engineering, Wuhan Polytechnic University, Wuhan, Hubei 430023, China and; CAS Key Laboratory of Computational Biology, CAS-MPG Partner Institute for Computational Biology, Shanghai Institute of Nutrition and Health, University of Chinese Academy of Sciences, Chinese Academy of Sciences, Shanghai 200333, P. R. China; CAS Key Laboratory of Computational Biology, CAS-MPG Partner Institute for Computational Biology, Shanghai Institute of Nutrition and Health, University of Chinese Academy of Sciences, Chinese Academy of Sciences, Shanghai 200333, P. R. China; CAS Key Laboratory of Computational Biology, CAS-MPG Partner Institute for Computational Biology, Shanghai Institute of Nutrition and Health, University of Chinese Academy of Sciences, Chinese Academy of Sciences, Shanghai 200333, P. R. China; Tianjin Institute of Industrial Biotechnology, Chinese Academy of Sciences, Tianjin 300308, P. R. China; CAS Key Laboratory of Computational Biology, CAS-MPG Partner Institute for Computational Biology, Shanghai Institute of Nutrition and Health, University of Chinese Academy of Sciences, Chinese Academy of Sciences, Shanghai 200333, P. R. China

## Abstract

Addition of chemical structural information in enzymatic reactions has proven to be significant for accurate enzyme function prediction. However, such chemical data lack systematic feature mining and hardly exist in enzyme-related databases. Therefore, global mining of enzymatic reactions will offer a unique landscape for researchers to understand the basic functional mechanisms of natural bioprocesses and facilitate enzyme function annotation. Here, we established a new knowledge base called EnzyMine, through which we propose to elucidate enzymatic reaction features and then link them with sequence and structural annotations. EnzyMine represents an advanced database that extends enzyme knowledge by incorporating reaction chemical feature strategies, strengthening the connectivity between enzyme and metabolic reactions. Therefore, it has the potential to reveal many new metabolic pathways involved with given enzymes, as well as expand enzyme function annotation.

**Database URL**: http://www.rxnfinder.org/enzymine/

## Introduction

Enzyme function annotation has excellent implications in metabolic engineering, synthetic biology and pathophysiology (1). Along with the rapid expansion of protein sequences, predicting enzymatic reactions of unannotated sequences using computational methodology is widely becoming used (2, 3). This function prediction contains enzyme feature extraction and classification optimization as two main procedures associated with machine learning and deep learning (4). Meanwhile, feature extraction is no longer limited to sequence similarity but includes more conservative features independent of sequence length (3), such as sequence patterns, structural features and catalytic mechanisms curated in PROSITE, CATH, SCOP and M-CSA (5–8). Sequence information curated in UniProt (9) acted as a vital resource for orphan sequence mining based on the assumption that similar sequences function consistently. In sequence-related features, PROSITE defines protein domains and sequence patterns of biological significance (6). Analysis of the structural domains generated by CATH reveals the prominent features of protein secondary structure (5). Enzyme 3D structural information from Protein Data Bank is also utilized to facilitate functional annotation beyond only sequence information (10). In terms of known structure, SCOP manually provides structural classification to reveal the evolutionary relationships between proteins (8). Structural information can help to identify binding sites and catalytic residues on proteins further. A specific collection of catalytic residues and detailed processes of catalytic mechanisms can be obtained from M-CSA (7). However, pieces of sequence- and structure-related feature information mentioned are scattered in each database, hindering the understanding and integration of enzyme annotation.

At the same time, chemical structure information dramatically improves the accuracy of model prediction and is considered indispensable for accurate prediction in the performance evaluation (11). Various annotation tools have applied reaction-based strategy to assign an Enzyme Commission (EC) number for reactions based on changes in the chemical structure of substrates and products (12–14). However, chemical structure information about enzymatic reactions is limited to compounds in reactions, lacking further feature mining. Currently, several integrated databases that provide detailed profiles about enzymes are available, each having a specific objective towards accelerating metabolic engineering and enzyme annotation. ExplorEnz offers a canonical curation of the International Union of Biochemistry and Molecular Biology (15). EzCatDB prompted a manual classification of enzyme reaction from the perspective of enzyme structure and catalytic mechanisms (16). BRENDA constructs a comprehensive enzyme information system with a focus on enzymatic reactions and relevant pathways (17). Based on enzyme and reaction databases, chemical transformation can be extracted, generalized for similar structural changes and applied to expand possible biosynthetic routes. BNICE defined generalized enzyme reactions for formulating enzyme reaction rules systematically; metabolic in silico network expansion and atlas of biochemistry were therefore depicted based on this computational framework (18–20). RetroRules took another approach to compute rules for available metabolic reactions that describe these reactions and can be plugged into RetroPath2.0 to design bioengineering pathways (21, 22). Reaction rule is gradually showing usefulness in exploring possible biosynthetic routes. However, these specific databases focus on reaction rules, lacking the interaction of enzyme information and place certain demands of chemical literacy on researchers. An enzymatic database with detailed enzyme information and intuitive chemical information extraction is urgently needed at present. A detailed comparison of the content of related databases is provided in Table 1.

**Table 1.** Detailed comparison of the content of databases

Database	Enzyme	Mechanism	Reaction	Chemical feature		
				Reaction centre	Reaction rule	Core-to-core
ExplorEnz (15)	√	—	—	—	—	—
EzCatDB (16)	√	√	√	—	—	—
BRENDA (17)	√	—	√	—	—	—
BNICE (24)	—	—	√	—	√	—
RetroRules (22)	—	—	√	—	√	—
EnzyMine	√	√	√	√	√	√

To conquer the deficiencies mentioned earlier, we proposed EnzyMine, a comprehensive enzyme feature and annotation database. First, to describe the diversity of enzyme data and provide a comprehensive enzyme feature, we collected sequence-structure-related features and catalytic features scattered in the above databases. Next, we replenished a systematic reaction feature mining of chemical structure. Reaction features are expanded beyond reaction compounds to further include reaction centre, reaction rule and core-to-core analysis using our previous work, Rxnblast (23). Core-to-Core is defined as the scaffold transformation made up of atoms that change during the reaction. In particular, core-to-core analysis can show the unique and complex structural scaffold change in an enzymatic reaction, helping to demonstrate the role of the enzyme on the molecular scaffold. This chemical feature mining can intuitively aid the understanding of chemical composition changes and reaction patterns.

In this report, we mined 7767 EC numbers and displayed these enzymes with comprehensive sequence and structural feature visually in 267 345 protein sequences across 8058 organisms, along with deep calculated chemical features in 9831 reactions. This study introduces the process of reaction feature mining and analysis, molecule structure-based searching methods and makes the following contributions: (i) curation of scattered enzyme sequence, structure, catalytic data; (ii) chemical feature mining and analysis of enzymatic reactions and (iii) efficient searching methods based on chemical structure query and text query.

## Materials and methods

### Data collection and database content

Using EC numbers in EXPASY release (2020/2/27) (ftp://ftp.expasy.org/databases/enzyme/enzyme.dat), EnzyMine displays 7767 enzymes with complete sequences, structural data and vital interface links to referenced databases. The databases include information on enzyme function, sequence information and family classification, as well as protein structure, molecular structure, known reactions and associated literature. Sources and the amount of available data are shown in Table 2.

**Table 2.** Current contents of EnzyMine

Related data	Description	Number
EC number	Enzyme commission number	7767
UniProt	Protein sequence data from UniProt (9)	267 345
Organism	Number of species under EC number	8058
PROSITE	Protein domains and functional sites in PROSITE (6)	1450
CATH	Protein superfamily classification (5)	2017
SCOP	Structural classification of protein (8)	9514
PDB	Tertiary structure data from Protein Data Bank (10)	141 414
Catalytic mechanisms	Detailed catalytic mechanisms of an enzyme in M-CSA (7)	864
Compound data	Compound data for substrates/products/cofactors	27 765
Reaction data	Enzymatic reactions with EC number in Rhea (16), KEGG (17)	9831
Reference	Reference about Enzyme and reaction	39 833

### Database architecture

**Figure 1.**
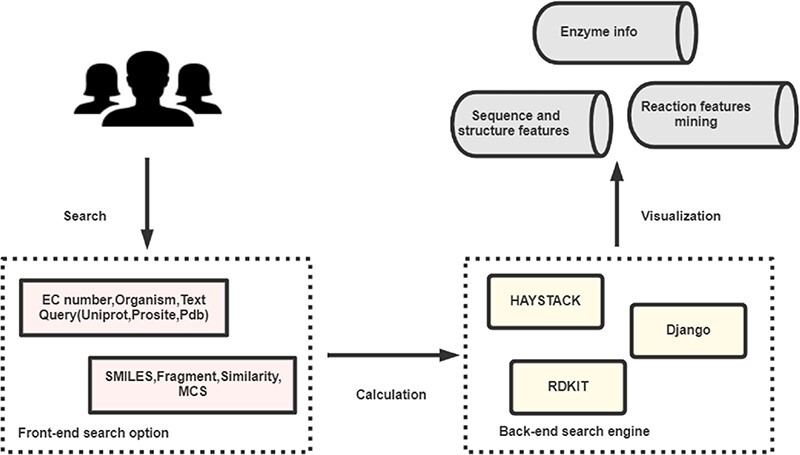
The broad architecture of EnzyMine processing, including the front-end and back-end search engines, which return displayed data.

EnzyMine is built on the Python web development framework, Django, with information stored in the PostgreSQL relational database. The front-end of the site is designed with HTML, CSS, Bootstrap, jQuery and JavaScript. Fuzzy search methods are completed by Haystack (http://haystacksearch.org/), an open-source and integrated full-text search engine in Django. Unique molecule searching methods are completed by RDKit (http://www.rdkit.org/), an open-source chemical informatics and machine learning kit that provides C++ and Python API interface. The architecture of the process is shown in Figure 1.

### The algorithm in query methods

Tanimoto coefficient performs remarkably well for similarity-based virtual screening by comparing chemical structures and is an appropriate choice for quantifying molecular similarity calculations. In particular, the Tanimoto coefficient-based similarity algorithm is used to construct a similarity retrieval method (24). Maximum common substructure (MCS) searching is established on the fMCS algorithm (25), providing a flexible and alternative powerful way to search by calculating the distance between two molecules. MCS percentage can be represented as a mathematical equation, T_MCS_ (A, B):

(1) \begin{equation*}{T_{MCS}}\left( {A,B} \right) = {{{{\left| {MCS\left( {A,B} \right)} \right|}_a}} \over {{{\left| A \right|}_a} + {{\left| B \right|}_a} + |MCS\left( {A,B} \right){|_a}}}\end{equation*}

In this equation, |A|a, |B|a represent the number of atoms for the query molecule and another molecule in our database, respectively. |MCS (A, B)|a, in this case, is the number of atoms in the MCS between the two molecules.

### Chemical feature extraction in an enzymatic reaction

**Figure 2.**
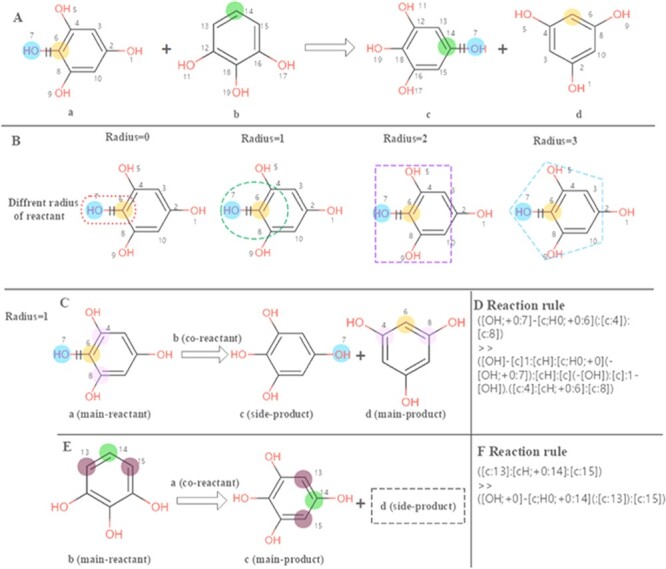
Calculation of the reaction rules for EC 1.97.1.2. (A) The reaction transformation is labelled. (B) Different radius can be used to identify adjacent neighbours in the reaction. (C, E) The reaction rules are calculated for two reactants. (D, F) reaction rules encoded with SMART.

The chemical feature mining strategy in EnzyMine is to perform regular extraction of enzymatic reactions, making great efforts in feature visualization, including reaction centre and scaffold transformation. In EnzyMine, we adopt SMARTS (SMILES arbitrary target specification), an expanded version of core-SMILES (simplified molecular-input line-entry system), as a code for reaction analysis (https://www.daylight.com/dayhtml/doc/theory/). Generally, atoms that change within a reaction are described as reaction centres, which are equivalent to atoms attached to the bonds that are broken or formed (21). Reaction rule summarizes the overall atom-bond pattern change taking place in the reaction and can be different according to the radius taken around the reaction centre (26). We used the RxnBLAST tool to extract the characteristics of reaction environment, including reaction centre, reaction rules and core-to-core, which focuses on extracting scaffold transformation and reactive chemical environment features by analysing atom-atom mapping (23). Our approach for visualizing the reaction centre and reaction rules is described in Figure 2. This generative process is divided into three parts. (1) The reaction centre is generated from atom-atom mapping. (2) Adjacent neighbours are characterized to the reaction centre using different radius; here, we choose a radius of 1 for specificity. (3) For multi-substrates/products reactions, reaction rules are extracted by assigning each reactant as the main reactant molecule at a time to limit the combinatorial complexity.

## Results

### Database overview

**Figure 3.**
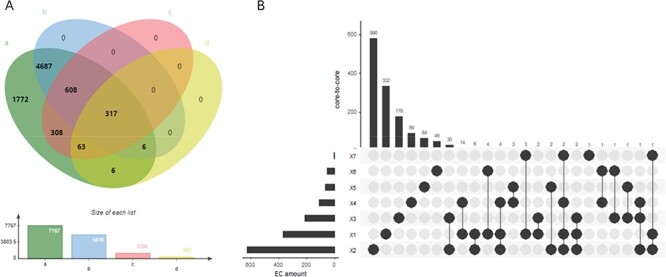
(A) Wynn diagram of four database coverage of EC number, a: sequence amount in UniProt, b: reaction amount in Rhea, c: family classification amount in PROSITE, d: 3D structure amount in Protein Data Bank. (B) The overall distribution of core-to-core across the seven types of enzyme.

EnzyMine displays 7767 enzyme items complemented with sequences, structural data, family classification, as well as molecular structure, known reactions and associated literature. Different coverage of EC number under each database is described in Figure 3A. With the rapid emergence of sequence data and improvement of sequencing methods, all the enzyme numbers are covered by sequencing data, while reaction data and family classification also reach a high coverage. At the same time, precise 3D structural data of enzymes still need to be revealed further by experimental studies.

At the reaction level, 9831 intuitive display results are found for the centre of enzyme-catalysed reactions, along with the extracted reaction rules and core-to-core characterization. In addition, in order to meet user’s design requirements of retrosynthesis, the website provides online calculation of reaction rules of different radii. Seven types of core-to-core distribution are shown in Figure 3B. In the distribution diagram, type two transferases react with a wide range of substrates, having the largest amount of core-to-core types. In addition, a small amount of core-to-core interaction can occur in the reaction across different types of enzymes, and this phenomenon occurs most frequently between the type two transferases and type three hydrolases.

### Search results and web usage

At the homepage, EnzyMine provides various search methods based on text and molecular retrieval algorithms When a user enters a query related to an ambiguous enzyme name and organism, the Haystack engine will show all possible results. Additionally, similarity and MCS searches for molecular queries will mainly return a series of outputs with similarity score/MCS percentage ranging from 1.0 or less. The detailed result is calculated using Equation (1). Compounds in the existing reactions can be searched by chemical structure or sequence similarity, and MCS processing provides metabolic results for many more molecules. At the all data page, we have listed the latest changes associated with each piece of data, such as the deletion of item 5.3.99.1, the transfer of 5.3.3.15 to 5.3.2.7 and the continuous increase of seven enzymes. Selecting an organism will return enzymes specific to that organism.

**Figure 4.**
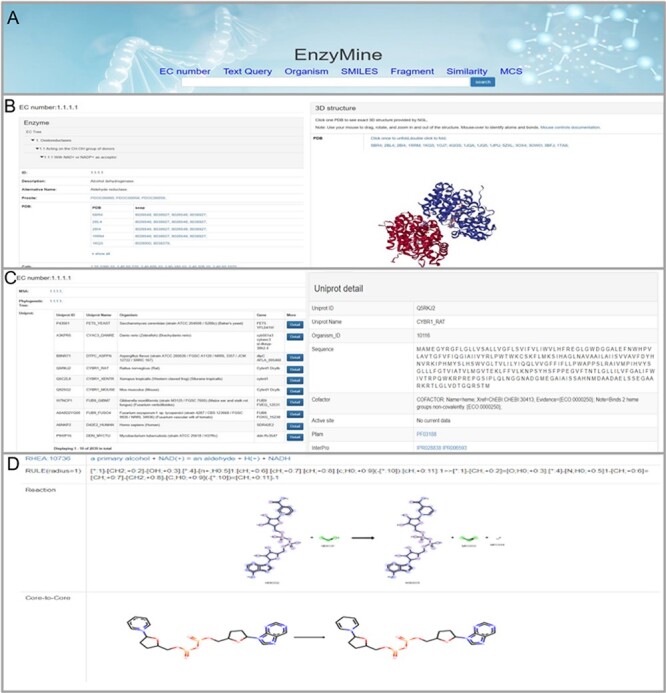
Detailed EnzyMine screenshot and search results for EC:1.1.1.1 are shown. (A) Front-end search methods based on text query and molecular searching query. (B) Basic enzyme information is listed, where EC classification, PROSITE and CATH classifications are shown on the left. Various display options related to protein 3D structures are provided by the NGL engine (27). (C) Multi-sequence alignment and phylogenetic trees related to the UniProt sequence under each the EC number are provided in a link, and single UniProt details are included by clicking the button on the right. (D) Reaction centre display, rules and core-to-core are shown. Detailed enzyme information and feature mining information are available for download.

These powerful search methods and result examples are shown in Figure 4A. On the page with details, the result is divided into three parts: Basic info, sequence analysis and reaction feature analysis. First, the basic info part displays enzyme information, 3D structures and related references, as shown in Figure 4B. Second, in sequence analysis, EnzyMine lists UniProt data in all organisms and function patterns in PROSITE under the EC number, as shown in Figure 4C. Multi-sequence alignment and phylogenetic tree facilitated sequence analysis help to provide a better understanding. Finally, 9831 calculated reaction centres, rules and core-to-core are provided in corresponding reaction analyses, as shown in Figure 4D. For enzymes with elucidated catalytic mechanisms in the sequence, we collected catalytic mechanisms from M-CSA, each connected to enzymatic reaction features shown by Sankey charts/Force-layout graphs. Force-layout graphs stress enzymatic catalytic features with active sites registered in UniProt and reaction under the EC number. Sankey diagrams offer more detail into the catalytic mechanism of each enzyme.

### Core-to-core analysis

**Figure 5.**
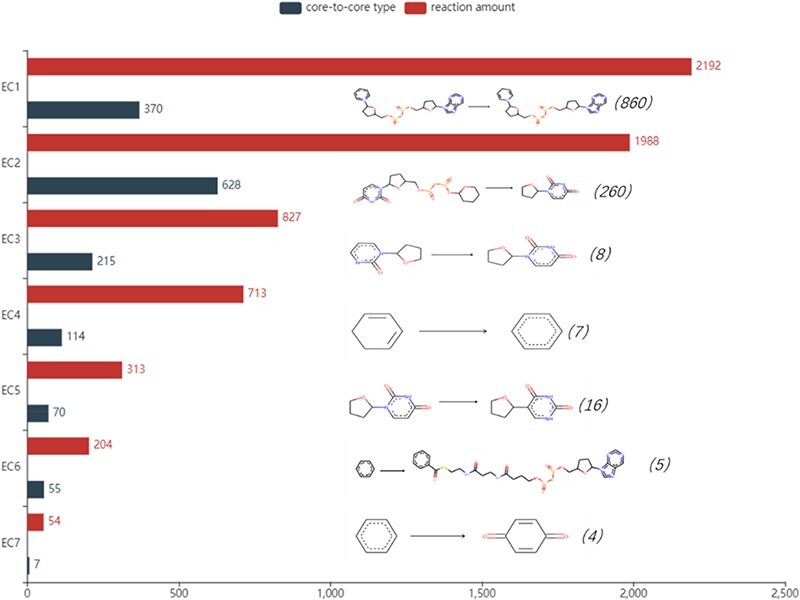
Detailed reaction amount and core-to-core type in seven types of enzymes, the most frequent amount marked in the parentheses and core-to-core pictures are listed on the right of the bar graph. EC1: Oxidoreductases, EC2: Transferases, EC3: Hydrolases, EC4: Lyases, EC5: Isomerases, EC6: Ligases, EC7: Translocases.

The number of reactions of seven enzymes and the analysis of core-to-core types are shown in Figure 5. The most frequent core-to-core type occurs in oxidoreductases, showing the process that NAD(+) takes hydrogen and turns into NADH in oxidoreductases. Transferases have the broadest range of the core-to-core type. Among these, the most numerous changes catalyse the transfer of glucuronic acid from Uridine disphosphate(UDP) to other (usually hydrophobic) molecules, which are quite common in membrane proteins to change the water solubility of the receptor molecules and promote the export of these molecules. In particular, a reaction with the rule may not have a core-to-core type. This phenomenon is relatively common in type 2 enzymes. For example, the reaction with Rhea ID 21824 belongs to transferase with EC number 2.6.1.1. It involves the conversion of side chains on the same skeleton, and core-to-core does not change before and after the reaction. All core-to-core data are available on the EnzyMine download page.

### Enzyme annotation integrations from sequence to reaction

Extended enzyme-number prediction method based on the core-to-core strategy is also available in EnzyMine as a simple application of those chemical feature data. EnzyMine can predict EC numbers in enzymatic reactions using the logic that different molecules with similar surroundings and the same reactive sites will share the same chemical transformations (28). It allows users to search for and annotate unannotated reactions. To enrich this annotation function for both sequence and reaction, a one-stop annotation integration is offered in the unfolded annotation tools on the EnzyMine homepage. EnzyMine utilizes the HMMER3 algorithm in FunFHMMer to complete the Gene Ontology annotation for protein sequences queries via a web server (29, 30). Bio2Rxn, a user-friendly platform for automatic reaction annotation based on protein sequences, has been integrated into EnzyMine ue to its consensus strategy and high precision in EC number prediction (31). It adopts the voting strategy to achieve high coverage and accuracy, which guarantees the *ab initio* understanding of enzymes and will expand the sequence annotation.

## Discussion

The collection of scattered feature data and supplementation of missing information are the direction in which database optimization is headed. Enzyme dataset provides the raw materials needed for insight into biological systems, but its potential can only be realized through high-level analysis. This would include feature exploration of unannotated sequences and reactions that lack protein sequence or identity. Textual description of the biochemical reaction in UniProt is now being replaced by the reaction in Rhea; high-quality biochemical information addition in enzyme annotation gradually shows its trend (32).

Conventional databases have, therefore, been expanded beyond sequence and structure data to include reaction centre visualization, reaction rule extraction and core-to-core analysis. Using current reaction data in EnzyMine, the prediction accuracy of returned EC number output can reach 92%, which can be improved further by a combination with enzyme sequence and structure data and prove the validity of chemical information. Reaction feature has the potential to provide more high-quality and low-latitude features for the feature extraction process of machine learning and is expected to improve the prediction accuracy of function annotation. In future studies, on the one hand, EnzyMine will continue to enrich data resources in line with data development. On the other hand, we will apply this high-valued chemical information to make contributions for further expansion of enzyme functional annotation.

## Conclusions

EnzyMine includes compiled and updated data integration in terms of enzyme and replenishes reaction chemical features. Reaction feature data in this study provide the visualization of reaction centre, a summary of reaction rules and analysis of core-to-core, which contributes to a deeper understanding of enzymatic reactions and will offer researchers a clearer view of biochemical processes and enzymative reactions that mediate them. In summary, the overall enzyme feature mining and holistic integration provide advanced and integrated resources to address enzyme function annotation.
